# Effects of different nitrogen doses and cultivars on fermentation quality and nutritive value of Italian ryegrass (*Lolium multiflorum* Lam.) silages

**DOI:** 10.5713/ab.21.0113

**Published:** 2021-06-23

**Authors:** Ibrahim Ertekin, Ibrahim Atis, Yusuf Ziya Aygun, Saban Yilmaz, Mustafa Kizilsimsek

**Affiliations:** 1 Department of Field Crops, Faculty of Agriculture, Hatay Mustafa Kemal University, Hatay 31060, Turkey; 2 Deparment of Field Crops, Faculty of Agriculture, Kahramanmaras Sutcu Imam University, Kahramanmaraş 46040, Turkey

**Keywords:** Annual Ryegrass, Fermentation Quality, Nitrogen Fertilizer, Nutritive Value, Silage

## Abstract

**Objective:**

The fermentation profile and silage quality of 3 Italian ryegrass (*Lolium multiflorum* Lam.) cultivars (cvs. Devis, Hellen, and Trinova) treated with 5 nitrogen doses (0, 50, 100, 150, and 200 kg/ha) were evaluated.

**Methods:**

The experiment was laid out in split plot in randomized complete block design with three replications. Annual ryegrass cultivars used in this study have been commonly grown in Turkey. Nitrogen doses were set in main plot and cultivars in split plot in the field. Plants were harvested at full-flowering stage with dry matter content about 220 g/kg for first cutting and 260 g/kg for second cutting. Harvested plants were chopped theoretically into 2 to 3 cm lengths for ensiling. Chopped fresh materials were ensilaged by compressing in 2 L plastic jars about 3±0.1 kg.

**Results:**

Effects of N doses on dry matter, neutral detergent fiber, acid detergent fiber, dry matter digestibility, relative feed value, crude protein, pH, ammonia nitrogen, lactic acid, acetic acid, and lactic acid/acetic acid were statistically significant while water soluble carbohydrate, ash and organic matter were not statistically different. Ammonia nitrogen, crude protein, ash, organic matter, lactic acid, and lactic acid/acetic acid were affected by cultivars, but the other parameters were not. Increasing nitrogen applications positively affected the chemical composition of annual ryegrass silage. The significant increase in protein content was remarkable, however, silage fermentation properties were adversely affected by the increasing nitrogen dose.

**Conclusion:**

It can be recommended 150 kg/ha nitrogen dose for annual ryegrass harvested at full blooming stage. Even though the silage fermentation properties of the used cultivars were similar, cv. Devis gave better results than the others in terms of silage pH and relative feed value.

## INTRODUCTION

Forage crop conservation is a significant subject in agricultural practices. One of the most common used techniques is ensiling method. Ensiling is based on natural fermentation in which the epiphytic lactic acid bacteria convert sugars into lactic acid under anaerobic conditions. Annual ryegrass with high forage quality is an important forage crop for animal feeding. This plant has been widely cultivated throughout temperate and tropical or subtropical regions of the world [1,2]. Also, the plant has been ensilaged in many countries and commonly used as a major silage crop.

Fertilization is the most effective treatment on yield and quality of plants and results of this practice are immediately ascertainable [3]. In addition, especially nitrogen fertilization increases quality of forage grasses significantly [4,5]. Many studies including nitrogen applications were carried out about forage yield and quality of Italian ryegrass. Even low nitrogen doses provide an increase in protein content and forage yield compared to the non-fertilizer treatments in Italian ryegrass. A rise in protein ratio is achieved after a certain nitrogen dose, but no increase in forage yield is observed [6]. Kunelius and Boswall [7] reported that the N fertilizer doses of 0.35 to 0.50 kg/ha with sowing, 0.35 to 0.50 kg/ha after first cutting and 0.65 to 0.80 kg/ha after second cutting were appropriate in annual ryegrass cultivation. Thereby, high forage yield and quality from Italian ryegrass can be obtained. It was determined that increasing the nitrogen fertilizer doses increased chlorophyll content of Italian ryegrass [8]. In addition, the highest crude protein (CP) and dry matter (DM) yield in Italian ryegrass were obtained from 120 kg/ha nitrogen treatment [9].

Many researchers reported that the plant yield and quality improved with the increasing nitrogen doses in Italian ryegrass cultivation. However, there is limited knowledge on the monitoring of the change in silage quality and ensilage performance of Italian ryegrass grown with increasing nitrogen doses. The goals of the current study were therefore to investigate the effects of different N treatments on silage fermentation quality and nutritive value of various Italian ryegrass cultivars.

## MATERIALS AND METHODS

### Planting, growing and silage preparation of annual ryegrasses

This study was carried out at Hatay Mustafa Kemal University in Turkey. Three different annual ryegrass (ARG) cultivars and nitrogen fertilizer were used as the material in this research. The experiment was laid out in split plot in randomized completed block design with three replications. ARG cultivars used in study have been commonly grown in Turkey. cvs Devis, Hellen and Trinova were obtained from Mutlu Seed, Alfa Seed and Semillas Fito Agriculture companies from Turkey. 0 (control), 50, 100, 150, and 200 kg/ha of nitrogen doses were set in main plot in experiment design and the ARG cultivars were sown in split plot. Fertilizer doses were applied to the soil surface in two parts, before planting and after first harvesting. ARG cultivars were planted in eight rows for each plot. Spaces of among the main plots, split plots and blocks were 1 m, 0.5 m and 3 m, respectively. ARG seeds were sown 50 kg/ha. ARG cultivars were planted in November 2017 with the first cutting in April 2018 and the second cutting in June 2018. Plants were harvested in full-flowering stage with DM content about 220 g/kg for first cutting and 260 g/kg for second cutting. Harvested fresh samples were chopped (CAN SP255, CANTEK MAKİNE, Sinop, Turkey) theoretically without wilting into 2 to 3 cm lengths to ensile. It was reserved about 500±20 g to determine the initial chemical compositions (Table 1) of materials from chopped plants. These samples were dried in an oven-drying cabinet at 65°C for 48 hours. Chopped fresh materials obtained from each plot were ensiled in 2 L plastic jars about 3±0.1 kg with 3 replications. Jars were stored at 25°C in dark conditions during the fermentation stage.

### Chemical analyzes and determination of nutritive value of silages

All silages were opened after 90 days’ fermentation. A sample of 20 g was taken from each silage and mixed with 180 mL of Ringer’s solution with a hand blender for 90 seconds at high speed and then filtered with Whatman 55 paper to obtain water extract [10]. pH measurements, water soluble carbohydrate (WSC), lactic acid (LA) and acetic acid (AA) and ammonia nitrogen (NH_3_-N) content were analyzed on silage water extract. pH measurements of silages were determined at the temperature of 25°C via pH meter (INOLAB, 8F93, Weilheim, Germany). The WSC contents of silages were analyzed according to the phenol-sulfuric acid calorimetric method reported by Dubois et al [11]. Samples of 300±20 g from silages were dried in the oven-drying at 65°C for 48 hours, ground in mill with 1 mm sieve diameter and made ready chemical analysis. Total N was determined according to method of 7.022 AOAC [12]. The CP contents were calculated with 6.25 coefficient unit. Ash contents were determined in an ash furnace by burning at 550°C for 4 hours. Cell wall contents such as neutral detergent fiber (NDF) and acid detergent fiber (ADF) were analyzed with ANKOM Fiber Analyzer (ANKOM Technology Corp., Fairport, NY, USA) according to the method described by AOAC [13]. Acid detergent lignin (ADL) analysis was made according to method reported by Robertson and Van Soest [14]. NH_3_-N contents of silages were determined with Kjehdahl distillation and titration apparatus according to method of Blümmel et al [15]. The LA and AA contents of silages were analyzed by using high performance liquid chromatography (HPLC, Shi-madzu GC-2010, Kyoto, Japan) at 42°C, 0.6 mL/min flow rate and by using refractive index detector described by Quiros et al [16] after the sample cleaning procedure. DM digestibility (DMD), DM intake (DMI) and relative feed value (RFV) properties of silages were calculated with the following formulas described by Van Dyke and Anderson [17].


$$\begin{array}{l} \text{DMD }(\%)=88.9-(0.779\times \text{ADF }\%)\\ \text{DMI }(\%)=120/\text{NDF }\%\\ \text{RFV}=\text{DMD }\%\times \text{DMI }\%\times 0.775 \end{array}$$

### Statistical analyzes

The statistical calculations of the data obtained this study were performed by split-split plot in randomized complete design using the statistical program of MSTAT-C. The general linear model was used to determine the differences among the means of investigated features in this study. Duncan multiple range test (p<0.05) was used to compare ARG cultivars and nitrogen doses and cutting ranks. In this study, interaction effects of ARG cultivars, nitrogen doses and cutting ranks used as experimental factors are not presented.

## RESULTS

### Fermentation features, chemical compositions and nutritive value of silages

The DM contents of annual ryegrass silages were decreased with increasing N doses. DM contents of silages were 23.0%, 25.3%, 23.2%, 20.8%, and 18.9% for the 0, 50, 100, 150, and 200 kg/ha N doses (N_0_, N_50_, N_100_, N_150_, and N_200_) respectively (Table 2). The DM contents of cultivars were not different. The effect of cutting rank on DM content was significant. The DM content obtained in the first cutting (18.8%) was lower than obtained in the second cutting (25.7%).

The pH values of annual ryegrass silages were significantly influenced by N dose. The lowest pH value was determined in the control treatment (N_0_) without nitrogen application. The pH values of N_50_, N_100_ and N_150_ treatments were statistically similar while the pH value of N_200_ treatment was significantly higher than others. No statistically significant difference was observed among the pH values of the cultivars. The effects of cutting ranks were significant in terms of the pH values, which were 4.79 and 4.57 for first and second cutting, respectively (Table 2).

NH_3_-N contents were influenced (p<0.01) by all experimental factors (Table 2). NH_3_-N contents of silages tended to increase with increasing N doses. However, the NH_3_-N contents determined in N_50_ and N_100_ nitrogen applications were not different from the control (N_0_). The NH_3_-N content increased significantly as the nitrogen dose increased to N_150_ kg/ha. The NH_3_-N content determined in N_200_ nitrogen application was higher than all other applications. The NH_3_-N contents ranged from 6.13% to 6.52% among the annual ryegrass cultivars. While cv. Devis and cv. Hellen had similar NH_3_-N content, NH_3_-N content of cv. Trinova was higher (p<0.01) than the others. NH_3_-N content determined as the first cutting (6.76%) was higher than determined at the second cutting (5.80%).

The effects of cutting rank were significant in terms of WSC while N doses and cultivars were insignificant (Table 2). WSC values were ranged among 4.30% and 6.72% depend on N doses. But these differences in values were statistically insignificant. WSC values recorded for cv. Devis, cv. Hellen and cv. Trinova were 5.92%, 4.88%, and 5.13%, respectively. WSC determined at the first cutting (6.52%) was higher (p< 0.01) than determined at the second cutting (4.10%).

The increase in nitrogen doses caused a decrease in NDF contents. NDF contents of silages were 66.4%, 66.9%, 66.0%, 62.4%, and 62.6% for N_0_, N_50_, N_100_, N_150_, and N_200_, respectively (Table 2). NDF contents of N_0_ and N_50_ were higher (p<0.01) than those of N_150_ and N_200_. The NDF content of N_100_ was similar to all other N applications. NDF contents of cultivars varied between 64.0% and 65.2%, but these differences were not significant. NDF percentage obtained at the first cut was significantly lower (p<0.05) than that obtained on the second cut (63.8% and 65.6%, respectively).

The effects of N doses were significant in terms of the ADF content. The change of ADF contents depending on the nitrogen doses showed a similar situation with the NDF contents. ADF contents ranged from 38.4% to 42.4% depending on N doses. The effects on ADF contents of cultivars and cutting ranks were not significant (Table 2). The ADF contents of the three cultivars were similar. ADF values determined in the first and second cuttings were also not different from each other.

The CP contents were significantly influenced by all experimental factors. A continuous increase in the CP content due to increased N doses was observed. The CP content values determined for each nitrogen dose application were significantly different. The CP contents of silages were 7.0%, 8.5%, 11.1%, 12.4%, and 13.0% for N_0_, N_50_, N_100_, N_150_, and N_200_, respectively (Table 3). Increasing the dose of nitrogen from N_0_ to N_200_ kg/ha resulted in an increase in CP content. The CP content of cv. Hellen was higher than other cultivars. The CP contents of cv. Devis and cv. Trinova were statistically similar (Table 3). The CP content of silage made at the first cutting (10.1%) was lower (p<0.001) than that of silage made at the second cutting (10.7%).

The effects of cultivars and cutting rank were significant in terms of the ash content while N doses had no significant effect (Table 3). The ash contents of silages were ranged from 11.7% to 13.1% depending on N doses. The highest ash content was obtained from cv. Devis. The ash contents of cv. Hellen and cv. Trinova were statistically similar. The ash content determined at the first cutting (13.8%) was higher than determined at the second cutting (12.5%).

Nitrogen had no significant effect on organic matter (OM) (Table 3). The OM content of cv. Devis (86.3%) was lower (p<0.01) than cvs. Hellen (87.3%) and Trinova (87.0%). The OM content of the first cutting was lower (p<0.01) than the OM content of the second cutting (86.3%).

The effects of N doses on DMI, DMD, and RFV were significant (p<0.01) and parallel. All three characteristics tended to increase with increasing N doses (Table 3). DMD, DMI, and RFV values determined in N_0_ and N_50_ doses were lower (p<0.05) than those of N_150_ and N_200_ while N_100_ was intermediate. DMD, DMI, and RFV values of N_100_ was similar to all other N applications. Cultivar had no significant effect on DMD, DMI, and RFV. DMI was decreased (p<0.05) at the second cut but no effects were noted on DMD or RFV. DMI value determined at the first cutting (1.9) was higher than determined at the second cutting (1.8).

A continuous decrease (p<0.01) in the LA content due to increased N was observed (Table 4). The highest LA content was obtained from control treatment (N_0_) while the lowest LA content was obtained from N_200_ nitrogen treatment. LA content of cv. Devis (3.46% DM) was lower (p<0.01) than cv. Hellen (3.64% DM) and cv. Trinova (3.65% DM). The LA was increased at the second cutting (p<0.01).

The trend of change of AA content depending on nitrogen doses was in the opposite direction of the trend of change in LA content. The AA content of silage DM increased from 1.14% at N_0_ to 1.66% at N_200_ (Table 4). Cultivar had no effect on AA content but AA content of the first cut (1.53% DM) was higher (p<0.01) than that observed in the second cut (1.30%).

## DISCUSSION

According to the results of the research, it can be said that forage quality characteristics (CP, NDF, ADF, DMD, DMI, and RFV) not related to ensiling were positively affected by increasing nitrogen doses, but characteristics related to silage (DM content, pH, NH_3_-N, LA content, AA content) are negatively affected by increasing nitrogen doses.

Increasing the dose of nitrogen to N_50_, N_100_, N_150_, and N_200_ kg/ha resulted in an increase of 21.8%, 58.5%, 77.3%, and 86.9% in CP content compared to the control, respectively. It can be said that the protein content of annual ryegrass increases in response to nitrogen application [18]. Similarly, it has been reported by other researchers that the protein content of annual ryegrass increases in response to nitrogen application [19]. Some researchers have reported higher %CP values for annual ryegrass than ours while other researchers have reported similar values to ours [19,20,21]. One of the main reasons for these differences may be that the harvest to obtain dry herbage is done at an earlier stage of plant maturity than the harvest for silage. Consistent with our findings, previous researchers determined that the protein contents of annual ryegrass silages varied between 6.33% and 14.54% [22,23]. Since protein is a major cost in supplements for livestock, the total amount of protein produced per unit area is one of the most important quality characteristics [24–26]. Therefore, nitrogen fertilization at the appropriate dose can be an effective tool to increase the protein content of low-protein grasses to meet the protein needs of animals. It was also determined that the NDF, ADF, DMD, DMI, and RFV values of the silages were positively affected by the increasing nitrogen doses. Our findings are inconsistent with some previous research results, which reported that these values increased or did not change with increasing nitrogen doses [18,27,28]. Pinho et al [29] reported that in terms of NDF, the response of pearl millet to nitrogen doses varies depending on genotypes. Cinar et al [22] reported that the effect on RFV of N doses changes depending on years. The effect of fertilization rate on grass degradability characteristics is not consistent [30].

Dry matter contents of annual ryegrass silages were decreased with increased N doses. With low N fertilization, DM contents of grasses increase [27] and this change in silage raw material is reflected in silage [29]. However, low DM content has a negative effect on the ensiling ability of the plant material. Dry matter content of forage crops at harvest is one of the most important factors for successful ensilage [31,32]. According to Castle and Watson [33] a minimal DM content of 247 g/kg is required for silage production. Therefore, excessive nitrogen fertilization in forage crops to be silage may pose a risk, especially in early mowing. The amount of LA produced by LA bacteria during silage fermentation is usually the acid concentration, and during the LA fermentation it is the acid that causes the greatest decrease in pH, 10 to 12 times stronger than AA found in silages [34]. The increase in pH, the amount of NH_3_-N and AA content due to the increase in nitrogen dose and the decrease in and LA content support this situation. Consistent with our results, Lv et al [4] reported that although high nitrogen application for ryegrass silage is an effective application in terms of nutrient content of silage, it may reduce the silage fermentation quality in silage, especially because it causes low DM content and high NH_3_-N content.

In the present study, DM content increased with advancement in cutting rank. This situation may be the result of high temperatures in the second cutting time. Similar differences in DM content at different cutting periods were reported by Valk et al [30] and Baldinger et al [23]. Baldinger et al [23] reported pH values close to our values while they determined higher LA values than our LA values for annual ryegrass. In the second cutting, better values were obtained in terms of ensiling properties (pH, NH_3_-N, LA, and AA) compared to the first cutting. This situation might be explained by increasing DM content [35]. Also, an increase in protein content was detected in the second cutting. This increase may have been due to nitrogen applied after first harvest. Also, Garcia del Moral et al [36] emphasized that forage CP content was negatively related to forage yield (data not shown) due to available N distributing in a greater volume of plant tissue.

Generally, quality parameters of the cultivars tested were similar. CP content and OM content of cv. Hellen were higher than for cvs Devis and Trinova. Our findings agree with Lale and Kökten [37], who reported that CP content changed depending on the cultivars. However, their calculated CP content for annual ryegrass was higher than our values. In another study, Colak and Sancak [20] reported that no significant difference in CP of annual ryegrass cultivars. In addition, Costa et al [38] determined that the CP content of varieties varied significantly depending on the plant growing stage. In our study LA content of cv. Devis was lower than cv. Hellen and cv. Trinova. There was no difference among cultivars in terms of other quality parameters examined. Our findings are consistent with Costa et al [38], who reported that the ADF and NDF contents of annual ryegrass harvested in the generative period were close to each other.

## IMPLICATIONS

Research results have shown that increasing nitrogen doses positively affect the chemical composition of annual ryegrass silage. Especially the significant increase in protein content was remarkable. However, silage fermentation properties were adversely affected by the increasing nitrogen dose. When these two data are evaluated together, 150 kg/ha nitrogen dose can be recommended for annual ryegrass harvested in full blooming stage. Cultivars tested in this study gave the similar results in terms of nutritive value and silage fermentation quality. In terms of silage fermentation values, especially considering the dry matter content and pH values, it can be said that the annual ryegrass produced from the second cutting is the better silage material.

## Figures and Tables

**Table 1:** Chemical composition and pH value of Italian ryegrass in response to nitrogen, cultivar and cutting rank

Item	DM (%)	pH	WSC (%)	NDF (%)	ADF (%)	CP (%)	Ash (%)	OM (%)
N Doses								
N_0_	23.6±1.11	6.00±0.02	13.1±1.04	62.2±0.82	36.0±0.38	8.5±0.21	11.7±0.25	88.3±0.25
N_50_	25.8±1.79	6.01±0.01	14.3±1.03	64.8±0.67	38.0±0.42	10.2±0.14	12.3±0.38	87.7±0.38
N_100_	23.7±1.64	6.03±0.02	13.3±1.36	64.1±0.93	37.7±0.71	11.2±0.16	12.8±.24	87.2±0.24
N_150_	21.4±1.30	6.05±0.01	9.6±1.22	64.2±0.70	37.2±0.52	13.0±0.30	12.3±0.32	87.7±0.32
N_200_	19.3±0.89	6.05±0.02	13.2±1.56	63.6±0.91	36.8±0.68	14.5±0.27	13.1±0.37	86.9±0.37
Mean	22.7	6.03	12.7	63.8	37.2	11.4	12.4	87.6
Cultivars								
Devis	23.3±1.23	6.04±0.01	11.1±0.86	65.4±0.55	37.9±0.48	11.3±0.42	12.4±0.23	87.6±0.23
Hellen	22.1±1.13	6.01±0.02	14.4±1.18	61.2±0.58	36.2±0.44	11.4±0.46	12.6±0.26	87.4±0.26
Trinova	22.8±1.04	6.02±0.01	12.6±0.85	64.6±0.51	37.4±0.36	11.7±0.39	12.3±0.27	87.7±0.27
Mean	22.7	6.03	12.7	63.8	37.2	11.4	12.4	87.6
Cutting rank								
1st	19.3±0.67	5.99±0.01	11.6±0.69	62.9±0.59	37.5±0.40	12.1±0.37	13.0±0.21	87.1±0.21
2nd	26.2±0.86	6.06±0.01	13.9±0.89	64.6±0.41	36.8±0.32	10.7±0.29	11.9±0.18	88.1±0.18
Mean	22.8	6.03	12.7	63.8	37.2	11.4	12.4	87.6

DM, dry matter; pH, power of hydrogen; WSC, water soluble carbohydrate; NDF, neutral detergent fiber; ADF, acid detergent fiber; CP, crude protein; OM, organic matter.

**Table 2:** Effects of N doses, cultivars and cutting rank on silage dry matter (DM), pH, NH_3_-N and some chemical compositions

Items	DM (%)	pH	NH_3_-N (% Total N)	WSC (% DM)	NDF (% DM)	ADF (% DM)
N Doses						
N_0_	23.0±1.07^ab^	4.47±0.05^c^	5.36±0.23^c^	4.30±0.56	66.4±0.71^a^	41.5±0.86^a^
N_50_	25.3±1.80^a^	4.61±0.04^b^	5.51±0.09^c^	5.38±0.82	66.9±0.91^a^	42.4±0.85^a^
N_100_	23.2±1.62^ab^	4.66±0.06^b^	5.53±0.13^c^	5.82±0.87	66.0±0.78^ab^	40.3±0.92^ab^
N_150_	20.8±1.30^bc^	4.71±0.04^b^	6.55±0.10^b^	4.32±0.53	62.4±0.78^b^	38.5±0.64^b^
N_200_	18.9±0.88^c^	4.96±0.05^a^	8.47±0.23^a^	6.72±0.61	62.6±0.76^b^	38.4±0.44^b^
Mean	22.2	4.68	6.28	5.31	64.7	40.2
p-value	^*^	^**^	^**^	ns	^*^	^*^
Cultivars						
Devis	21.6±1.24	4.72±0.06	6.13±0.30^b^	5.92±0.62	64.0±0.85	40.1±0.69
Hellen	22.4±1.11	4.67±0.04	6.21±0.20^b^	4.88±0.51	65.0±0.71	40.0±0.71
Trinova	22.7±1.02	4.65±0.04	6.52±0.23^a^	5.13±0.51	65.2±0.47	40.4±0.55
Mean	22.2	4.68	6.28	5.31	64.7	40.2
p-value	ns	ns	^**^	ns	ns	ns
Cutting rank						
1st	18.8±0.65^b^	4.79±0.04^a^	6.76±0.20^a^	6.52±0.46^a^	63.8±0.64^b^	40.4±0.63
2nd	25.7±0.85^a^	4.57±0.03^b^	5.80±0.19^b^	4.10±0.36^b^	65.6±0.44^a^	39.4±0.37
Mean	22.2	4.68	6.28	5.31	64.7	39.9
p-value	^**^	^**^	^**^	^**^	^*^	ns

DM, dry matter; pH, power of hydrogen; NH_3_-N, ammonia nitrogen; WSC, water soluble carbohydrate; NDF, neutral detergent fiber; ADF, acid detergent fiber; ns, non-significant.

a–c Mean values with different superscripts have significant differences.

** p<0.01;

* p<0.05.

**Table 3:** Effects of N doses, cultivars and cutting rank on silage some chemical compositions and nutritive value

Items	CP (% DM)	Ash (% DM)	OM (% DM)	DMD (%)	DMI	RFV
N Doses						
N0	7.0±0.16^e^	12.7±0.40	87.3±0.40	56.6±0.67^b^	1.8±0.02^b^	79.5±1.63^b^
N50	8.5±0.09^d^	13.1±0.33	86.9±0.33	55.9±0.66^b^	1.8±0.02^b^	78.1±1.88^b^
N100	11.1±0.17^c^	13.1±0.28	86.9±0.28	57.5±0.72^ab^	1.9±0.02^ab^	82.6±1.92^ab^
N150	12.4±0.10^b^	13.4±0.29	86.6±0.29	58.9±0.50^a^	1.9±0.02^a^	88.2±1.70^a^
N200	13.0±0.17^a^	13.3±0.29	86.7±0.29	59.0±0.34^a^	1.9±0.02^a^	88.0±1.56^a^
Mean	10.4	13.1	86.9	57.6	1.9	83.3
p-value	^**^	ns	ns	^*^	^*^	^*^
Cultivars						
Devis	10.3±0.40^b^	13.7±0.24^a^	86.3±0.24^b^	57.6±0.54	1.9±0.02	84.3±1.81
Hellen	10.6±0.45^a^	12.7±0.26^b^	87.3±0.26^a^	57.7±0.56	1.9±0.02	82.9±1.60
Trinova	10.3±0.46^b^	13.1±0.21^b^	87.0±0.21^a^	57.4±0.43	1.9±0.01	82.6±1.11
Mean	10.4	13.1	86.9	57.6	1.9	83.3
p-value	^**^	^**^	^**^	ns	ns	ns
Cutting rank						
1st	10.1±0.36^b^	13.8±0.19^a^	86.3±0.19^b^	57.0±0.49	1.9±0.02^a^	83.8±1.52
2nd	10.7±0.35^a^	12.5±0.16^b^	87.5±0.16^a^	58.2±0.29	1.8±0.01^b^	82.8±0.91
Mean	10.4	13.1	86.9	57.6	1.9	83.3
p-value	^**^	^**^	^**^	ns	^*^	ns

CP, crude protein; OM, organic matter; DMD, dry matter digestibility; DMI, dry matter intake; RFV, relative feed value; ns, non-significant.

a–e Mean values with different superscripts have significant differences.

** p<0.01;

* p<0.05.

**Table 4:** Effects of N doses, cultivars and cutting rank on some silage products and LA/AA

Items	Lactic acid (% DM)	Acetic acid (% DM)	LA/AA
N Doses (kg/ha)			
N_0_	4.18±0.11^a^	1.14±0.04^e^	3.83±0.25^a^
N_50_	3.73±0.09^b^	1.27±0.04^d^	2.99±0.15^b^
N_100_	3.48±0.06^c^	1.44±0.03^c^	2.43±0.07^c^
N_150_	3.40±0.05^c^	1.56±0.04^b^	2.20±0.07^c^
N_200_	3.13±0.05^d^	1.66±0.04^a^	1.91± 0.07^d^
Mean	3.58	1.41	2.67
p-value	^**^	^*^	^**^
Cultivars			
Devis	3.46±0.10^b^	1.41±0.05	2.56±0.19^b^
Hellen	3.64±0.08^a^	1.43±0.04	2.70±0.13^ab^
Trinova	3.65±0.08^a^	1.41±0.05	2.76±0.17^a^
Mean	3.58	1.42	2.67
p-value	^**^	ns	^*^
Cutting rank			
1st	3.36±0.04^b^	1.53±0.03^a^	2.25±0.07^b^
2nd	3.80±0.08^a^	1.30±0.03^b^	3.09±0.15^a^
Mean	3.58	1.42	2.67
p-valure	^**^	^**^	^**^

DM, dry matter; LA/AA, lactic acid/acetic acid; ns, non-significant

a–e Mean values with different superscripts have significant differences.

** p<0.01;

* p<0.05.
